# Six Months Follow-Up of Patients with Invasive Mechanical Ventilation Due to COVID-19 Related ARDS

**DOI:** 10.3390/ijerph18115861

**Published:** 2021-05-29

**Authors:** Ayham Daher, Christian Cornelissen, Niels-Ulrik Hartmann, Paul Balfanz, Annegret Müller, Ingmar Bergs, Maria Aetou, Nikolaus Marx, Gernot Marx, Tim-Philipp Simon, Dirk Müller-Wieland, Bojan Hartmann, Alexander Kersten, Tobias Müller, Michael Dreher

**Affiliations:** 1 Department of Pneumology and Internal Intensive Care Medicine, University Hospital RWTH, 52074 Aachen, Germany; ccornelissen@ukaachen.de (C.C.); amueller@ukaachen.de (A.M.); ibergs@ukaachen.de (I.B.); maetou@ukaachen.de (M.A.); tobmueller@ukaachen.de (T.M.); mdreher@ukaachen.de (M.D.); 2 Department of Cardiology, Angiology and Internal Intensive Care Medicine, University Hospital RWTH, 52074 Aachen, Germany; nihartmann@ukaachen.de (N.-U.H.); pbalfanz@ukaachen.de (P.B.); nmarx@ukaachen.de (N.M.); dirmueller@ukaachen.de (D.M.-W.); bhartmann@ukaachen.de (B.H.); akersten@ukaachen.de (A.K.); 3 Department of Intensive Care and Intermediate Care, University Hospital RWTH, 52074 Aachen, Germany; gmarx@ukaachen.de (G.M.); tsimon@ukaachen.de (T.-P.S.)

**Keywords:** coronavirus, critical illness, follow-up, pulmonary function, fatigue, depression, anxiety, quality of life

## Abstract

Although patients who recovered from acute coronavirus disease 2019 (COVID-19) may have prolonged disabilities, follow-up data of those who have survived COVID-19 related acute respiratory distress syndrome (ARDS) is still very scarce. Therefore, COVID-19-ARDS survivors requiring invasive mechanical ventilation (IMV) were followed six months after discharge. Pulmonary function tests (PFTs), 6-min walk test (6MWT) and echocardiography were performed. Quality of life (QoL), depression and anxiety were assessed using validated questionnaires. Patients were compared based on respiratory mechanics and CT-phenotype during intensive care unit (ICU) stay. Eighteen patients were included (61 ± 7 years; ICU-stay: 34 ± 16 days; IMV: 30 ± 15 days). At follow-up (197 ± 15 days after discharge), PFTs did not reveal significant limitations (VC: 92 ± 16%; FEV1: 92 ± 20%; DLco/VA: 81 ± 16%). Cardiac systolic function was normal in all patients, but 50% of them had diastolic dysfunction. 6MWT was under the lower limit of normal in only two patients. Eight patients (44%) reported tiredness, six (33%) suffered from fatigue and one patient (6%) had depression and anxiety. Surprisingly, patients with worse respiratory mechanics during IMV reported fewer symptoms and less exertional dyspnea at follow-up. In conclusion, patients with COVID-19-ARDS have the possibility to fully recover regarding pulmonary function and exercise capacity, which seems to be independent of disease severity during ICU stay.

## 1. Introduction

As more than a year has passed since the beginning of coronavirus disease 2019 (COVID-19) pandemic, a growing population of individuals has recovered from severe acute respiratory syndrome coronavirus type 2 (SARS-CoV-2) infection. However, a growing body of evidence suggests that these patients may experience a wide range of symptoms and abnormalities after recovery from acute illness, which may persist for more than several months [1]. These observations led to the emergence of the new terms “long COVID” [1], and “post COVID syndrome” [2]. Currently, it is not clear whether these abnormalities are unique to COVID-19, how long they persist and how they develop further. The course of long term “post COVID” abnormalities in patients who had been treated with invasive mechanical ventilation (IMV) in an intensive care unit (ICU) is even less well described.

The 6-month follow-up data after hospital discharge from Huang et al. showed that fatigue was the main symptom of patients who had recovered from COVID-19 and that impairments in pulmonary diffusion capacity were more pronounced in patients after more severe illness [3]. However, in the Huang’s study all patients hospitalized with severe disease were lumped together, and only ten of them had been treated with IMV or extracorporeal membrane oxygenation (ECMO) during their hospital stay [3].

The aim of the current study was to investigate symptoms, abnormalities in pulmonary function, the prevalence of non-pulmonary organ dysfunctions and psychological disorders in patients who had been treated with IMV due to COVID-19 ARDS six months after discharge from hospital. These data could provide a better understanding of this emerging disease and could help to ensure an adequate and timely management of significant health limitations with the aim of restoring premorbid quality of life (QoL) [4].

## 2. Materials and Methods

The present prospective study included 18 consecutive patients who had been hospitalized during the first COVID-19 wave between 24 February 2020 and 21 April 2021 at the university hospital RWTH Aachen due to COVID-19 and who were admitted to the ICU needing IMV due to acute respiratory distress syndrome (ARDS). SARS-CoV-2 infection was confirmed by reverse-transcriptase–polymerase-chain-reaction (RT-PCR) in a respiratory tract sample. During the aforementioned period, 56 patients were admitted to the ICU with COVID-19, of whom 26 did not survive to hospital discharge. The presented survivors were managed with follow-up appointments in the pulmonary disease outpatient clinic six months after discharge.

The protocol for this study was approved by the local ethics committee (EK 080/20). All investigations were performed in accordance with the ethical standards of the latest revision of the Helsinki Declaration. Written informed consent was obtained from all patients, their legal representative in cases of severe consciousness disorders, or the consulting physician, if appropriate. Written informed consent was obtained from all patients as early as possible.

Regarding assessment during ICU stay, patients were categorized regarding the severity of ARDS according to the “Berlin Definition” on the day of intubation [5]. Laboratory parameters, arterial blood gas analysis (ABG), total respiratory compliance (Crs) and ventilation variables including partial pressure of oxygen/fraction of inspired oxygen (P/F) ratio were extracted from the patient data management system after inclusion in the study. Depending on respiratory mechanics and radiologic features two primary “phenotypes” of the pulmonary disease were identified as described previously: Type L, characterized by low elastance (i.e., high compliance), low ventilation-to-perfusion ratio, low lung weight and low recruitability; and Type H, characterized by high elastance, high right-to-left shunt, high lung weight and high recruitability [6]. Further data regarding renal failure, liver failure, ICU length of stay and length of IMV were recorded.

At follow-up, full pulmonary function tests (PFTs), electrocardiography and transthoracic echocardiography were performed. Furthermore, serum, plasma and whole blood samples were obtained. Among other tests, a complete blood count and coagulation studies including D-dimer levels were performed, as well as the measurement of serum levels of C-reactive protein (CRP), different cytokines, N-terminal pro B-type natriuretic peptide (NTproBNP), creatine kinase (CK), high sensitive cardiac troponin T (hs-cTnT) and creatinine. Furthermore, health-related QoL was assessed. With support of a trained study team, patients answered different clinical questionnaires to assess various aspects of their quality of life including: Patient Health Questionnaire 9 (PHQ-9) of depression [7], Generalized Anxiety Disorder 7 (GAD-7) (on both scales, minimal symptoms are represented by a score of 0–4, mild symptoms by a score of 5–9, moderate symptoms by a score of 10–15 and severe symptoms by a score ≥15) [8], St. George’s Respiratory Questionnaire (SGRQ) (which is scaled from 0 representing optimal health to 100 reflecting worst health, and has three main components: symptoms component evaluates respiratory symptoms, activities component evaluates the physical activity, and the impacts component assesses social and psychological limitations) [9,10] and EQ-5D-5L (Euro Quality of Life–Five Dimensions–Five Levels) questionnaire, which is a descriptive system that defines health in terms of 5 dimensions: mobility, self-care, usual activities, pain/discomfort and anxiety/depression; each with 5 levels (Level 1: no problems, 2: slight problems, 3: moderate problems, 4: severe problems and 5: unable to/extreme problems) [11]. For comparison of the SGRQ results, normal values for the European population from the large Spanish study of Ferrer et al. were used [12].

Whole-body plethysmography (MasterLab, Viasys, Hoechberg, Germany) was performed before and after bronchodilation (including DLco measurement only after bronchodilation) according to current guidelines and recommendations [13,14,15]. Samples for ABG were taken from the arterialized earlobes of all patients while breathing room air without supplemental oxygen (ABL 800 flex, Radiometer, Copenhagen, Denmark).

All patients underwent the 6-min walk test (6MWT) without supplemental oxygen, with measurements of vital signs including oxygen saturation (SpO2) and Borg-scale before and after exercise according to current recommendations [16,17,18]. The difference of the actual walking distance to the predicted value and the lower limit of normal (lower 95% confidence interval) were calculated for all patients.

Statistical analyses were performed using standard descriptive statistics including mean ± standard deviation, median (interquartile range), frequencies and percentages (%). Between-group differences were tested using Kruskal–Wallis test and χ2 test for continuous and categorical variables, respectively. Nominal *p* values are presented.

## 3. Results

Until 21 April 2021 a total 30 patients were discharged from hospital after being treated with IMV due to severe COVID-19 with ARDS. Until 22 March 2021; 18 of them (age 61 ± 7, 61% male) were evaluated in the pulmonary disease outpatient clinic six months after discharge from the ICU. Baseline characteristics, medical history and characteristics during ICU stay are described in Table 1.

### 3.1. Patients’ Characteristics during ICU Stay

All patients suffered from severe COVID-19 with ARDS and were managed with invasive mechanical ventilation in ICU. Of these, 5 patients (28%) had severe ARDS according to Berlin-Definition and 13 patients (72%) had moderate ARDS. The mean ICU length of stay and mean length of IMV were 34 ± 16 days and 30 ± 15 days, respectively. Two patients received extracorporeal membrane oxygenation (ECMO) therapy with a mean duration of the ECMO run of 13 ± 4 days. Seven patients (39%) developed acute renal failure requiring continuous renal replacement therapy.

Further, 13 patients (72%) had type L pneumonia (respiratory compliance in first week 50 ± 21 mL/cmH_2_O) and 5 patients (28%) had type H (respiratory compliance 25 ± 11 mL/cmH_2_O). Additionally, 13 patients (72%) needed prone position.

Patients were discharged from hospital after a mean of 44 ± 16 days. Further demographic data as well as data regarding in-hospital treatment are demonstrated in Table 1.

### 3.2. Six-month Follow Up Data

Patients were seen in our outpatient clinic for a follow-up examination about six months (197 ± 15 days) after hospitalization. Table 2 shows symptoms of patients at the follow-up visit. The most frequent symptoms were tiredness (44%) and fatigue (33%), whereas respiratory symptoms such as dyspnea and coughing were reported less frequently (17%).

In the whole group, PFTs including ABG revealed no impairments (total lung capacity (TLC): 94 ± 11% of predicted; vital capacity (VC): 92 ± 16%; forced expiratory volume in 1 s (FEV1): 92 ± 20%; FEV1/forced vital capacity (FEV1/FVC): 81 ± 9%; diffusion capacity/alveolar volume (DLco/VA) 81 ± 16%; partial pressure of oxygen (PaO2): 72 ± 10 mmHg; partial pressure of carbon dioxide (PaCO2): 37 ± 4 mmHg) (Table 3). Nevertheless, four patients had abnormal PFTs: an obstructive pattern was observed in one patient with known chronic obstructive pulmonary disease (COPD), a restrictive pattern was observed in three patients of whom one had been diagnosed with restrictive respiratory disorder due to neuromuscular disease prior to COVID-19 and two had only a mild restriction which was not previously known (VC: 71% and 86%; TLC: 81% and 75%; FEV1: 92% and 97%; DLco/VA: 72% and 84%, respectively). In summary, only two patients showed impairments in PFTs at 6-month follow-up which could be attributed to COVID-19, and these impairments were mild. Low dose CT of the latter two patients showed mild subpleural “fibrous stripes” predominantly in the lower lobes. Another four patients had an isolated reduction in diffusion capacity (DLCO/VA < 80%), whereas only one of them suffered from exertional dyspnea, which was unchanged compared to the time before COVID-19.

In the 6MWT, despite slightly reduced minute walk distances (6MWD) under predicted values in 13 patients, the vast majority (89%) had 6MWD above their age-adjusted lower limit of normal (6MWD median = 483 m (IQR 403–536), difference to the predicted value = −36 m (−115–0) and difference above the predicted LLN = +108 m (+31–+146)). However, there was no significant drop in oxygen saturation after exercise in any patient (SpO2 after exercise: 95 ± 3%).

Transthoracic echocardiography revealed no abnormalities in left or right ventricular systolic function. However, 50% of patients had diastolic dysfunction though there was no information available whether this was a pre-existing condition, but 11 of 18 (61%) patients had hypertension in their medical history (Table 3).

Laboratory parameters at hospital admission and at follow-up are represented in Table 4. At hospital admission, patients had increased D-dimer levels (median = 2349 ng/mL (IQR: 1406–4607)), and increased serum lactate dehydrogenase (LDH) activity (median = 444 U/L (IQR: 402–681)), as well as high inflammatory parameters (CRP, ferritin and IL-6). Furthermore, hs-cTnT and CK values were elevated. At the time of follow up, all abovementioned laboratory parameters were in the normal range.

According to PHQ-9 and GAD-7 questionnaires, only one patient had severe depression and symptoms of anxiety, and the majority had minimal to mild symptoms (Table 5). The SGRQ showed mainly reduced physical activity (activity score: 37.2 ± 35.1). QoL scores at follow-up are represented in (Table 5).

When comparing patients with and without fatigue, no significant differences were found with respect to preexisting conditions or other variables.

### 3.3. Comparison of Patients with Type H versus Type L Pneumonia

Table 1, Table 2, Table 3, Table 4 and Table 5 stratify out results by the phenotype of type H and type L pneumonia. Patients with type H pneumonia had more acute illness. They arrived at the hospital earlier than those with type L (admission at 2 ± 3 vs. 9 ± 5 days after symptom onset, respectively, *p* = 0.02), ended up earlier in intensive care unit (6 ± 4 vs. 12 ± 4 days after symptom onset, respectively, *p* = 0.04) and required earlier intubation (7 ± 3 vs. 12 ± 4 days, respectively, *p* = 0.03) (Table 1). There were no significant differences between the two groups regarding medical history and other baseline characteristics (Table 1). In line with the definition of the phenotypes, respiratory compliance stayed significantly lower in the H compared to the L group (respiratory compliance in the first week after intubation: 25 ± 11 vs. 50 ± 21 mL/cmH_2_O respectively, *p* < 0.01). Furthermore, patients in the H group had a tendency to higher morbidity, with a lower P/F ratio at admission, longer ICU stay and longer IMV duration. ECMO was exclusively used in H-type patients. However, none of these differences reached statistical significance (Table 1).

Interestingly, there were no significant differences in PFTs between the two groups at follow-up; in fact, PFTs including DLco were slightly better in the H group compared to the L group (Table 3). Furthermore, H patients had less dyspnea after exercise than L patients (dyspnea after 6MWT on Borg scale: 1 ± 1.5 vs. 3 ± 2.8, respectively, *p* < 0.05). Using QoL scores, H patients tended to have better scores especially regarding SGRQ scores, though these differences did not reach statistical significance (Table 5).

## 4. Discussion

This study has shown that pulmonary recovery is possible in patients who survived COVID-19 associated ARDS, independent from disease severity and respiratory mechanics during ICU stay. Pulmonary function tests including exercise tests were not significantly impaired at follow-up regarding the whole study group. In addition, there were no abnormalities in cardiac systolic function. However, some patients were still symptomatic six months after discharge from hospital with fatigue and tiredness being the most frequent symptoms.

Many observational studies have reported that hospitalized patients with COVID-19 have prolonged disabilities and may not return to previous levels of work by 6 months after acute infection [2,3]. However, follow-up data of COVID-19 patients who have been treated with mechanical ventilation is very scarce to date, which is mainly owed to the long term in-hospital management and the need of prolonged post-discharge rehabilitation of these patients [19]. However, this group requires particular attention due to the burden of mechanical ventilation (e.g., ventilator-induced lung injury), as well as possible sequelae of critical illness and its management in ICU. A significant proportion of patients in our cohort reported fatigue and tiredness at follow-up (33% and 44%, respectively). Surprisingly, the prevalence of these symptoms in our cohort was lower than the previously reported prevalence after 6 months with up to 66% in other cohorts of patients not requiring IMV [3]. Furthermore, the proportion of patients suffering from fatigue and tiredness after ICU discharge in our cohort is similar to previous studies on ICU patients prior to the COVID-19 era [20,21]. In summary, our findings suggest that COVID-19 patients requiring therapy in an ICU over a long period of time, do not have an over-proportional susceptibility to post-COVID symptoms compared with COVID-19 patients with milder disease and even compared to other patient groups requiring long term treatment including invasive ventilation in ICU. The analysis of Qol scores in our cohort shows that patients´ limitations are largely due to limitations in the ability to be mobile and active, and are not owed to psychological disorders such as depression or stress.

Data on PFTs following COVID-19 are evolving [3], but once again there are few data on critically ill patients after invasive ventilation. To the best of our knowledge, this is the first study to show that despite long ICU stay with a long duration of ventilation, and poor respiratory mechanics, patients had no significant PFT abnormalities at follow-up. Even patients with previously documented reduced lung function had no deterioration in pulmonary function at follow-up. In fact, there was a small proportion of patients who had a reduction in diffusion capacity, which is consistent with the conclusions of previous studies on other COVID-19 cohorts [3,22]. However, there were no correlations between PFT abnormalities and symptoms at follow-up.

Similar to other in-hospital COVID-19 scenarios [23], patients in our study had normal right and left ventricular systolic function assessed by transthoracic echocardiography. However, the prevalence of left ventricular diastolic dysfunction was high. The clinical significance of the last finding needs to be addressed in future studies, as many observational studies suggest a relationship between COVID-19 and heart failure with preserved ejection fraction (HFpEF) [24]. Although case reports have described profound COVID-19 myocarditis leading to HFpEF, the more common manifestation in the COVID-19 era may be HFpEF related primarily to the unmasking of subclinical HFpEF and secondarily to the development of new HFpEF following infection with SARS-CoV-2 [24]. Furthermore, 11 of 18 (61%) patients in our cohort had hypertension (Table 3), which is probably the single most common cause of diastolic dysfunction and HFpEF overall.

Previous studies have surprisingly shown that the occurrence of post-COVID sequelae and pulmonary abnormalities are not associated with disease severity [25], which can be confirmed by the findings in our study, so that one can conclude that critical ill COVID-19 patients are not at a higher risk to post-COVID symptoms or cardiopulmonary abnormalities.

As patients with type H and type L pneumonia represent two different categories in many aspects during management in ICU, comparing long-term follow-up parameters of these two groups might be of clinical importance in order to further manage those patients. As expected, H patients in our cohort had more severe disease, worse respiratory mechanics and longer ICU stay during acute COVID-19. Surprisingly, at 6-month follow-up the latter patients tended to have less symptoms, less exertional dyspnea and better activity scores. In addition, cardiac function did not differ between patients with type L and H pneumonia. These findings support our conclusion that disease severity in the ICU does not predict the long term “post-COVID” course of the disease.

Our study has some limitations which need to be addressed. First, the number of investigated patients was quite low. Secondly, due to the small number it is not possible to evaluate the effects of various therapies during ICU stay on the post-COVID course. Nevertheless, these preliminary data might help to design future studies to better understand the sequelae of COVID-19 on the long term in order to improve patients’ quality of life.

## 5. Conclusions

In conclusion, patients with ARDS due to COVID-19 have the possibility to fully recover with regard to their pulmonary function and exercise capacity, and this seems to be independent of disease severity and respiratory mechanics during ICU stay. Fatigue and tiredness are the most prominent symptoms in these patients, while psychological disorders such as depression and anxiety do not seem to contribute to patient limitations.

## Figures and Tables

**Table 1:** Baseline characteristics, medical history and characteristics during ICU stay.

	All Patients (*n* = 18)	Type H (*n* = 6)	Type L (*n* = 12)	*p*-Value
Age, years	61 ± 7	60 ± 10	61 ± 7	1.00
Female, *n* (%)	7 (39)	4 (67)	3 (25)	0.14
**Comorbidities, *n*** (**%**)				
COPD	3 (17)	1 (17)	2 (17)	1.00
Bronchial asthma	2 (11)	1 (17)	1 (8)	1.00
Hypertension	11 (61)	2 (33)	9 (75)	0.14
Heart Failure	1 (6)	0 (0)	1 (6)	1.00
Atrial fibrillation	1 (6)	0 (0)	1 (8)	1.00
Chronic kidney disease	2 (11)	0 (0)	2 (17)	1.00
Coronary artery disease	3 (17)	1 (17)	2 (17)	1.00
Diabetes mellitus	2 (11)	1 (17)	1 (8)	1.00
**Symptom onset to, days**				
Hospitalization	7 ± 6	2 ± 3	9 ± 5	0.02
Intensive care admission	10 ± 5	6 ± 4	12 ± 4	0.04
Intubation	11 ± 4	7 ± 3	12 ± 4	0.03
**In-hospital Periods, days**				
Fever days	30 ± 12	31 ± 18	29 ± 10	0.91
Hospital length of stay	44 ± 16	50 ± 20	40 ± 14	0.33
Oxygen supplementation	39 ± 18	47 ± 23	35 ± 14	0.26
**Characteristics during ICU stay**				
P/F Ratio at ICU admission	143 ± 53	107 ± 47	162 ± 48	0.06
Mean of P/F Ratio after 50% of the total duration of ventilation	267 ± 81	293 ± 135	259 ± 61	0.90
Total respiratory compliance in the first week, mL/cmH_2_O	42 ± 22	25 ± 11	50 ± 21	<0.01
Total respiratory compliance in the third week, mL/cmH_2_O	56 ± 37	28 ± 11	70 ± 38	0.07
Duration of ICU stay, days	34 ± 16	40 ± 22	31 ± 12	0.45
Duration of Ventilation, days	30 ± 15	34 ± 19	27 ± 12	0.61
Patients on ECMO, *n* (%)	2 (11)	2 (33)	0 (0)	0.10
Duration of ECMO, days	13 ± 4	13 ± 4	-	-
Prone position, *n* (%)	13 (72)	5 (83)	8 (66)	0.61
Continuous neuromuscular blockade (NMB) > 6 h at any point, *n* (%)	3 (17)	0 (0)	3 (25)	0.51
CRRT, *n* (%)	7 (39)	1 (17)	6 (50)	0.32
Antibiotic therapy, *n* (%)	17 (94)	6 (100)	11 (92)	1.00
**Discharge to**				
Rehabilitation, *n* (%)	6 (33)	2 (33)	4 (33)	-
Other hospital, *n* (%)	2 (11)	1 (17)	1 (8)	-
Home, *n* (%)	10 (56)	3 (50)	7 (58)	-
**Time from discharge to follow-up, days**	197 ± 15	198 ± 5	196 ± 18	0.96

Values are presented as mean ± standard deviation or number of patients (percentage). COPD = chronic obstructive pulmonary disease; CRRT = continuous renal replacement therapy; ECMO = Extracorporeal membrane oxygenation; ICU = intensive care unit; P/F = partial pressure of oxygen/fraction of inspired oxygen.

**Table 2:** Symptoms and clinical examination at follow-up after 6 months.

	All Patients (*n* = 18)	Type H (*n* = 6)	Type L (*n* = 12)	*p*-Value
**Examination and vital parameters**				
Height, cm	175 ± 9	170 ± 8	177 ± 9	0.08
Weight, kg	91 ± 17	83 ± 13	95 ± 18	0.19
BMI, kg/m^2^	30 ± 6	30 ± 8	30 ± 6	0.89
Respiratory rate, bpm	17 ± 3	17 ± 2	17 ± 4	0.68
Oxygen saturation, %	98 ± 1	98 ± 1	98 ± 2	0.44
Oxygen flow, l/min	0 ± 1	0 ± 0	0 ± 1	0.56
Temperature, °C	36.5 ± 0.2	36.5 ± 0.2	36.5 ± 0.3	0.81
Systolic BP, mmHg	135 ± 18	144 ± 19	130 ± 17	0.12
Diastolic BP, mmHg	86 ± 20	78 ± 33	90 ± 10	0.78
Heart rate, bpm	77 ± 12	76 ± 9	77 ± 13	0.51
Frailty Score	4 ± 2	4 ± 2	4 ± 1	0,72
**Symptoms, *n*** (**%**)				
Tiredness	8 (44)	2 (33)	6 (50)	0.64
Fatigue	6 (33)	1 (17)	5 (42)	0.60
Headache	5 (28)	1 (17)	4 (33)	0.61
Rhinorrhea	4 (22)	2 (33)	2 (17)	0.57
Dyspnea	3 (18)	0 (0)	3 (25)	0.51
Myalgia	3 (18)	1 (20)	2 (17)	1.00
Cough	3 (17)	1 (17)	2 (17)	1.00
Angina pectoris	3 (17)	0 (0)	3 (25)	0.51
Loss of Taste	3 (17)	1 (17)	2 (17)	1.00
Sore throat	2 (11)	0 (0)	2 (17)	0.53
Cognitive disorders	2 (11)	0 (0)	2 (17)	0.53
Loss of Smell	2 (11)	0 (0)	2 (17)	0.53
Fever	0 (0)	0 (0)	0 (0)	1.00
Hemoptysis	0 (0)	0 (0)	0 (0)	1.00
Pharyngalgia	0 (0)	0 (0)	0 (0)	1.00
**Gastrointestinal symptoms, *n*** (**%**)				
Diarrhea	0 (0)	0 (0)	0 (0)	-
Nausea	1 (6)	0 (0)	1 (8)	1.00
Emesis	1 (6)	0 (0)	1 (8)	1.00
Stomach pains	0 (0)	0 (0)	0 (0)	-

Values are presented as mean ± standard deviation or number of patients (percentage). BMI = body-mass-index; BP = blood pressure.

**Table 3:** PFTs, 6MWT and echocardiography at follow-up after 6 months.

	All Patients (*n* = 18)	Type H (*n* = 6)	Type L (*n* = 12)	*p*-Value
**Pulmonary function parameters and ABGs**				
TLC, % of predicted	94 ± 11	92 ± 9	95 ± 12	0.62
VC, % of predicted	92 ± 16	95 ± 6	91 ± 19	0.92
RV, % of predicted	104 ± 24	93 ± 20	110 ± 25	0.27
RV/TLC, % of predicted	103 ± 22	96 ± 16	107 ± 25	0.42
FEV1, % of predicted	92 ± 20	99 ± 8	89 ± 23	0.42
FEV1/FVC, %	81 ± 9	84 ± 3	79 ± 10	0.06
Reff, % of predicted	84 ± 24	88 ± 29	82 ± 23	0.76
DLCO, % of predicted	65 ± 16	70 ± 9	62 ± 18	0.57
DLCOc/VA, % of predicted	81 ± 16	89 ± 6	78 ± 18	0.23
PaO2, mmHg	72 ± 10	75 ± 11	70 ± 10	0.31
PaCO2, mmHg	37 ± 4	38 ± 3	36 ± 4	0.31
pH	7.43 ± 0.05	7.42 ± 0.02	7.43 ± 0.06	0.76
Base excess, mmol/l	0.01 ± 2.60	0.06 ± 1.44	−0.02 ± 3.13	0.55
**6MWT**				
Distance, m	463 ± 134	438 ± 140	476 ± 135	0.81
SpO2 before exercise, %	96 ± 2	95 ± 2	97 ± 2	0.07
SpO2 after exercise, %	95 ± 3	95 ± 3	96 ± 3	0.81
HR before exercise, bpm	78 ± 15	85 ± 16	75 ± 14	0.28
HR after exercise, bpm	90 ± 21	94 ± 13	88 ± 25	0.61
Dyspnea on Borg scale before exercise	1.1 ± 1.3	0.4 ± 0.8	1.5 ± 1.4	0.11
Dyspnea on Borg scale after exercise	2.6 ± 2.6	1.1 ± 1.5	3.3 ± 2.8	<0.05
Fatigue on Borg scale before exercise	1 ± 1	1 ± 1	1 ± 2	0.31
Fatigue on Borg scale after exercise	3 ± 2	2 ± 1	3 ± 3	0.92
**Echocardiography**				
LVEF > 50%, *n* (%)	18 (100)	6 (100)	12 (100)	1.00
Diastolic dysfunction (Grade I-IV), *n* (%)	9 (50)	3 (50)	6 (50)	1.00
RVEF–normal, *n* (%)	15 (83)	4 (67)	11 (92)	0.25
TAPSE, mm	20 ± 6	19 ± 10	21 ± 4	0.72
RVSP + CVP, mmHg	28 ± 10	26 ± 9	30 ± 11	0.70

Values are presented as mean ± standard deviation or number of patients (percentage). ABGs = arterial blood gases; BP = blood pressure; CVP = central venous pressure; DLco = diffusing capacity for carbon monoxide; FEV1 = forced expiratory volume in 1 s; FVC = forced vital capacity; HR = heart rate; LVEF = left ventricular ejection fraction; 6MWT = six minute walk test; PaCO2 = partial pressure of carbon dioxide; PaO2 = partial pressure of oxygen; PFTs = pulmonary function tests; Reff = effective specific resistance; RV = residual volume; RVEF = right ventricular ejection fraction; RVSP = right ventricular systolic pressure; SpO2 = oxygen saturation; TAPSE = tricuspid annular plane systolic excursion; TLC = total lung capacity; VA = alveolar volume; VC = vital capacity.

**Table 4:** Laboratory findings of the whole cohort at admission day in ICU and at follow-up.

	Reference Values	Admission Day (*n* = 18)	Follow Up (*n* = 18)
**Hematology**			
White blood cells, 1/nL	4.0–10.0	9.2 (6.7–10.3)	6.5 (5.55–7.725)
Hemoglobin, g/dL	m: 14.0–18.0	12.2 (9.5–13.3)	14.45 (13.375–15.6)
	w: 12.0–16.0		
Platelets, 1/nL	150–400	234.5 (205–363)	256.5 (216–287.5)
Lymphocytes, %	22.0–53.0	10.6 (6.1–15.1)	27.15 (22.625–31.375)
**Coagulation**			
D-dimer, ng/mL	<500	2349 (1406–4607)	279 (225–544)
**Clinical Chemistry**			
AST, U/L	<35	70.5 (41.5–105.3)	24.5 (19.25–26.75)
ALT, U/L	<35	45 (20–61)	21 (17.25–30)
Gamma-GT, U/L	<40	58 (32.5–92.5)	36 (17.5–59)
LDH, U/L	m: 135–225	444 (402–681)	194.5 (175.75–216.25)
	w: 135–214		
CK, U/L	m: <174	193.5 (95.5–718.8)	91 (82–133.25)
	w: <140		
hs-Troponin T, pg/mL	<14.0	18.5 (14.5–23.3)	8.5 (6–14)
NTproBNP, pg/mL	<220	179.8 (131.1–261.7)	110.9 (33.15–177.325)
Creatinine, mg/dL	0.5–1.2	1.1 (0.8–1.5)	0.975 (0.875–1.1675)
CRP, mg/L	<5	172.7 (117.5–269.1)	1.9 (1.05–2.725)
PCT, ng/mL	<0.5	0.5 (0.2–5.3)	0.055 (0.03–0.08)
**Iron metabolism**			
Ferritin, ng/mL	15.0–150.0	776 (589.5–4687)	29.25 (24.225–42.3)
**Cytokines**			
sIL-2-receptor, U/mL	158–623	-	489 (353–635)
IL-6, pg/mL	<7.0	117.4 (103.4–214.3)	2.75 (1.97–4.325)

Values are median (interquartile range). ALT = alanine transaminase; AST = aspartate transaminase; CK = creatine kinase; CRP = C-reactive protein; Gamma-GT = gamma-glutamyltransferase; hs-Troponin-T = high sensitive troponin-T; IL-6 = interleukin-6; LDH = lactate dehydrogenase; NT-proBNP = N-terminal pro B-type natriuretic peptide; sIL2 = soluble interleukin-2 receptor; TNF = tumor necrosis factor.

**Table 5:** Questionnaires at follow up.

	All Patients (*n* = 18)	Type H (*n* = 6)	Type L (*n* = 12)	*p*-Value
**PHQ-9**	6 ± 5	4 ± 3	8 ± 6	0.17
Minimal to mild depression, *n* (%)	13 (72%)	6 (100%)	7 (58%)	-
Moderate depression, *n* (%)	4 (22%)	0 (0%)	4 (33%)	-
Severe depression, *n* (%)	1 (5%)	0 (0%)	1 (8%)	-
**GAD-7**	4 ± 4	2 ± 3	4 ± 5	0.23
Minimal to mild anxiety, *n* (%)	16 (89%)	6 (100%)	10 83%)	-
Moderate anxiety, *n* (%)	1 (5%)	0 (0%)	1 (8%)	-
Severe anxiety, *n* (%)	1 (5%)	0 (0%)	1 (8%)	-
**SGRQ**				
Symptoms Score (normal < 16.13 ± 16.77)	24.9 ± 23.6	12.8 ± 18.6	30.9 ± 24.1	0.11
Activity Score (normal < 16.28 ± 20.41)	37.2 ± 35.1	18.1 ± 33.8	46.8 ± 33.0	0.11
Impacts Score (normal < 8.14 ± 14.12)	17.7 ± 23.1	6.3 ± 13.2	23.9 ± 25.4	0.10
Total Score (normal < 12.17 ± 14.89)	24.8 ± 26.2	11.0 ± 20.1	32.3 ± 26.8	0.10
**EQ-5D-5L**				
Mobility (walking) score	2 ± 1	2 ± 1	2 ± 1	0.84
Severe to very severe mobility problem, *n* (%)	3 (17%)	1 (17%)	1 (17%)	-
Self-Care score	2 ± 1	2 ± 1	2 ± 1	0.71
Severe to very severe mobility problem, *n* (%)	0 (0%)	0 (0%)	0 (0%)	-
Usual Activities score	2 ± 1	2 ± 1	2 ± 1	0.88
Severe to very severe self-Care problem, *n* (%)	0 (0%)	0 (0%)	0 (0%)	-
Pain/Discomfort score	2 ± 1	2 ± 1	2 ± 1	0.67
Severe to very severe pain/Discomfort problem, *n* (%)	1	0 (0%)	0 (8%)	-
Anxiety/Depression score	1 ± 1	1 ± 1	1 ± 1	0.80
Severe to very severe anxiety/Depression problem, *n* (%)	0 (0%)	0 (0%)	0 (0%)	-
EQ VAS score	64 ± 21	68 ± 18	62 ± 23	0.67

Values are presented as mean ± standard deviation or number of patients (percentage). EQ-5D-5L = Euro Quality of Life–Five Dimensions–Five Levels; GAD-7 = Generalized Anxiety Disorder 7; PHQ-9 = Patient Health Questionnaire 9; SGRQ = St. George’s Respiratory Questionnaire.

## Data Availability

Data are available upon reasonable request to the corresponding author.
